# In Situ Construction of Elastic Solid-State Polymer Electrolyte with Fast Ionic Transport for Dendrite-Free Solid-State Lithium Metal Batteries

**DOI:** 10.3390/nano14050433

**Published:** 2024-02-27

**Authors:** Jin Wang, Yunlong Liao, Xi Wu, Lingfeng Ye, Zixi Wang, Fugen Wu, Zhiping Lin

**Affiliations:** 1 School of Materials and Energies, Guangdong University of Technology, Guangzhou 510006, China; wangjin_em@163.com (J.W.); vasiliki@yeah.net (X.W.); 2 The College of Information Engineering, Guangzhou Vocational University of Science and Technology, Guangzhou 510550, China; 3 School of Physics and Optoelectronic Engineering, Guangdong University of Technology, Guangzhou 510006, China; 15338525971@163.com (Y.L.); 19874208682@163.com (L.Y.); zixiwang2022@163.com (Z.W.)

**Keywords:** elastic solid-state polymer electrolyte, in situ polymerization, high room-temperature ionic conductivity, all-solid-state batteries, electrochemical properties

## Abstract

Solid-state lithium metal batteries (LMBs) have been extensively investigated owing to their safer and higher energy density. In this work, we prepared a novel elastic solid-state polymer electrolyte based on an in situ-formed elastomer polymer matrix with ion-conductive plasticizer crystal embedded with Li_6.5_La_3_Zr_1.5_Ta_0.5_O_12_ (LLZTO) nanoparticles, denoted as LZT/SN-SPE. The unique structure of LZT/SN-SPE shows excellent elasticity and flexibility, good electrochemical oxidation tolerance, high ionic conductivity, and high Li^+^ transference number. The role of LLZTO filler in suppressing the side reactions between succinonitrile (SN) and the lithium metal anode and propelling the Li^+^ diffusion kinetics can be affirmed. The Li symmetric cells with LZT/SN-SPE cycled stably over 1100 h under a current density of 5 mA cm^−2^, and Li||LiFePO_4_ cells realized an excellent rate (92.40 mAh g^−1^ at 5 C) and long-term cycling performance (98.6% retention after 420 cycles at 1 C). Hence, it can provide a promising strategy for achieving high energy density solid-state LMBs.

## 1. Introduction

Rechargeable lithium-ion batteries with organic electrolytes play a crucial role in modern society. However, several technical challenges, including low energy density and safety concerns, still need to be addressed to meet the demand for future applications [1,2,3]. To overcome these issues, solid-state LMBs, which are composed of lithium metal anodes and solid-state electrolytes, were developed and became an important way to achieve a new energy storage system with high safety and high energy density [4,5,6]. As the key part of solid-state LMBs, a wide variety of solid-state electrolytes were extensively studied [7,8,9,10,11,12]. Compared with mechanically hard and fragile inorganic solid-state electrolytes, solid-state polymer electrolytes (SPEs) are usually flexible with better interface compatibility and facile processability [13,14,15]. Polyethylene oxide (and its derivatives)-based SPEs are the most extensively investigated type of polymer electrolytes but are limited by insufficient ionic conductivity, a narrow electrochemical window (lower than 4 V), and the lack of effectively preventing the growth of Li dendrites due to their low Young’s modulus [14,16]. Therefore, many efforts were made to enhance the ionic conductivity and mechanical properties from the design of new types of solid electrolytes, the optimization of polymer matrix materials, and the regulation of polymer electrolyte components [9,15,17,18,19,20].

SN (N≡C-CH_2_-CH_2_-C≡N), as a notable plasticizer crystal, possesses ultra-high ionic conductivity of 1.1 mS cm^−1^ at 20 °C, which is much superior to most conventional SPEs [21,22]. Therefore, SN was used as an electrolyte additive, a plasticizer for polymer electrolytes, and for SN-based electrolytes due to its properties of non-flammability and high ionic conductivity [23,24,25,26]. However, the mechanical strength of SN-based SPEs decreases with an increase in SN content [27]; also, SN shows high activity with a lithium metal anode that leads to side effects [7,28]. The solid-state LMBs with SN-based SPEs exhibited poor electrochemical performance and could only cycle at low current densities [28]. Therefore, a novel SN-based SPE configuration should be designed to increase the content of SN whilst retaining good mechanical properties. Moreover, the stability of a lithium anode with SN and the ion conduction capability should be further improved for better rate capability of the solid-state LMBs.

Recently reported works provided promising solutions to the above-mentioned issues with in situ phase separation concepts [18,29]. Using this method, SN can be adopted as the main ion-conducting phase, while the three-dimensional interconnected elastic polymer phase can act as the elastomeric agent, the latter of which can be tutored with changes to the polymer species [30,31]. In the meantime, many studies were conducted to alleviate the side effects of SN and lithium metal, including introducing additives such as fluoroethylene carbonate (FEC) and Li-salt to construct a protective solid-electrolyte interphase (SEI) layer in situ [28,32]. However, the spontaneously generated SEI layer cannot effectively protect the lithium anode and their cycle performances are still unsatisfactory at high current densities.

Constructing composite polymer solid electrolytes with inorganic nanofillers is the most welcomed method to improve the ionic conductivity, electrochemical stability, and mechanical strength of SPEs [33,34,35,36]. The addition of ion-conducting nanofillers, such as Li_3x_La_2/3−x_TiO_3_ (LLTO), Li_7_La_3_Zr_2_O_12_ (LLZO), and LLZTO, can further enhance the Li^+^ conduction of SPEs [37]. Compared with LLTO and LLZO, LLZTO possesses higher ionic conductivity and less side reaction, which is the reason it is selected here as the representative inorganic additive [38,39,40]. The addition of LLZTO can not only enhance the mechanical strength in SN-based SPEs but also improve the electrochemical properties such as ionic conductivity and ion migration [41,42]. What is more, the La atoms of LLZTO can impose a strong coordination interaction between the nitrile groups of SN, which can protect the lithium anode from an attack by the free SN molecules [14,43]. At present, it is still a significant challenge to achieve a stable interface between the lithium metal anode and the high proportion SN-based SPEs and simultaneously maintain the available mechanical strength of the electrolyte.

Herein, we develop an elastic solid-state polymer electrolyte with the in situ construction of a 3D interconnected ion-conducting plastic crystal phase (SN) and inorganic LLZTO ceramic particles into an elastomer polymer matrix (butyl acrylate, BA), denoted as LZT/SN-SPE. The in situ construction of the SN phase with high ion conductivity can facilitate fast Li-ion conduction, and the elastic polymer phase possesses excellent elasticity and flexibility and can maintain intimate contact with the electrode during repeated cycling. More importantly, the addition of LLZTO can not only accelerate fast ion conduction and enhance the mechanical properties but can also act as the free-SN anchoring agent to eliminate the side reaction with the lithium anode. The LZ/SN-PSE exhibits high room temperature ionic conductivity (1.69 mS·cm^−1^) and excellent elasticity, enabling dendrite-free solid-state LMBs with enhanced rate capabilities. Stable Li plating/stripping behavior in the Li||Li symmetric cells is achieved at the current density of 1 mA cm^−2^ and 5 mA cm^−2^. What is more, the solid-state LMBs based on a LiFePO_4_ (LFP) cathode also deliver outstanding rate performance with a discharge capacity of 92.40 mAh g^−1^ at 5 C and long-term cycling lifespan with 98.6% capacity retention after 420 cycles at 1 C. Therefore, this elastomeric polymer solid electrolyte can provide a promising strategy for achieving high energy density solid-state LMBs.

## 2. Methods

### 2.1. Preparation of Samples

The LZT/SN-SPE is prepared in four steps. Firstly, the LLZTO-BA-based solutions are prepared by dissolving 1 mol% poly(ethylene glycol) diacrylate (PEGDA, average Mn ~575), 1 M d lithium bis(trifluoromethanesulfonyl)imide (LiTFSI), and 10 wt% LLZTO ceramic particles in BA liquid. The mixture is further stirred for 12 h at room temperature. Secondly, SN-based solutions are prepared by mixing SN and 1 M LiTFSI at 70 °C for 20 min. Then, 5 vol% FEC additive is added to the mixture to prevent side reactions of SN with lithium metal and stirred for 1 h at 50 °C. Thirdly, LLZTO-BA/SN precursor solutions are prepared by mixing each mixture solution in a volume ratio of 1:1 at 50 °C and stirring for 30 min, then 0.5 mol% azobisisobutyronitrile (AIBN) is added and stirred for 15 min. Finally, prepared LLZTO-BA/SN precursor solutions are polymerized at 70 °C for 2 h to form the LZT/SN-SPE. As a comparison, the same experimental procedure is used without adding LLZTO to prepare the BA/SN solid-state polymer electrolyte (SN-SPE). All the processes are completed in an argon-filled glovebox.

### 2.2. Material Characterization

Scanning electron microscopy (SEM) is carried out using TESCAN MIRA LMS (TESCAN, Brno, The Czech Republic) to characterize the morphologies of samples. X-ray diffraction (XRD) is carried out using Ultima-IV (Origin Systems, Austin, TX, USA) to identify the phase structure of samples. Fourier transform infrared (FTIR) spectra are obtained on a Niolet iN10 spectrometer (Thermo Fisher Scientific). X-ray photoelectron spectroscopy (XPS) spectra data are obtained on Thermo Scientific K-Alpha+ (Thermo Fisher Scientific, Waltham, MA USA). Thermogravimetric analysis testing (TGA) is measured using Discovery TGA 550 (TA Instruments, New Castle, DE, USA) from room temperature to 800 °C at a heating rate of 10 °C min^−1^ under a nitrogen atmosphere. The uniaxial tensile tests are conducted on Inspekt Table Blue 5KN testing machine (Hegewald & Peschke, Nossen, Germany). Samples are cut into the size of 150 mm × 15 mm × 1.25 mm and tested with a crosshead speed of 30 mm min^−1^. The lithium metal electrodes used for SEM and XPS characterization are cleaned with DME several times and dried in an Ar-filled glove box.

### 2.3. Preparation of Cathode 

The LFP cathode is prepared as following steps: commercial LFP powders, conductive carbon black powders (Super P), polyvinylidene difluoride (PVDF) and SN-LiTFSI (molar ratio of 20:1) with a weight ratio of 7:1:1:1 are dispersed in N-methyl-2-pyrrolidone (NMP) and stirred for 12 h to form a homogeneous slurry. Then, the slurry is coated onto the aluminum foil coated by carbon and transferred into a vacuum oven at 50 °C for 12 h. The as-prepared cathode is punched into discs with a diameter of 10 mm and the areal loading of active material is about 1–1.5 mg cm^−2^.

### 2.4. Solid-State Batteries Assembling and Testing

The electrochemical properties of the elastic solid-state polymer electrolyte are investigated using CR2032 coin cells assembled in an Ar-filled glovebox. For the assembly process, the LFP cathodes are used as working electrodes lithium metal is used as the anode, and Celgard 3500 (Celgard LLC, Concord, NC, USA) is used as the separator. The precursor is injected into the separator and the cells are packaged immediately. After that, the assembled cells are heated at 70 °C for 2 h to generate the in situ polymerized LZT/SN-SPE (or SN-SPE). Due to the liquid nature of the precursor, Celgard 3500 separators are applied to all cells to prevent a short circuit in the assembled cells before polymerization. The charge/discharge experiments are carried out on a Neware Battery system with the potential ranges of 2.5–4 V at a current density of 1 C. Li||Li symmetric cells are prepared with an identical method except that LFP is replaced by Li foils (diameter of 12 mm). The Li plating/stripping experiments are measured on a Neware Battery system. 

### 2.5. Electrochemical Characterization and Analysis

The ionic conductivity (σ) of the electrolytes is measured using SS||electrolyte||SS symmetric cells with electrochemical impedance spectroscopy (EIS) test from 100 kHz to 1 Hz with an amplitude of 10 mV at the open-circuit potential. The ionic conductivity is tested at different temperatures in the range of 30~80 °C and is calculated based on the Equation (1):

(1) $$\sigma =\frac{L}{R\cdot S}$$

where R is the bulk resistance of the electrolyte measured by EIS and L and S are the thickness and surface area of the electrolyte membrane. The activation energy (*E_a_*) is derived from the slope of the Arrhenius plot. The Li-ion transference number (*t_+_*) is determined from alternating-current impedance and direct-current polarization measurements using Li||Li symmetric cells. Alternating-current impedance is conducted on a frequency range from 100 kHz to 0.01 Hz with an amplitude of 10 mV at open-circuit voltage.

(2) $$t_{Li^{+}}=\frac{I_{S}}{I_{0}}[\frac{\Delta V-I_{0}R_{0}}{\Delta V-I_{s}R_{s}}]$$

where ∆*V* (10 mV in this study) is the constant applied voltage during polarization, *I*_0_ and *I_s_* are the initial and steady-state currents during polarization, and R_0_ and R_s_ are the initial resistance and steady-state resistance before and after the polarization. Linear-sweep voltammetry (LSV) is carried out using Li||stainless steel asymmetric cells from 1.5 V to 6 V versus Li/Li^+^. The sweeping rate is 1 mV s^−1^. All cells are assembled in an argon gas-filled glove box and electrochemical tests are using the Shanghai Chen Hua electrochemical station.

## 3. Results and Discussion

**Figure 1:**
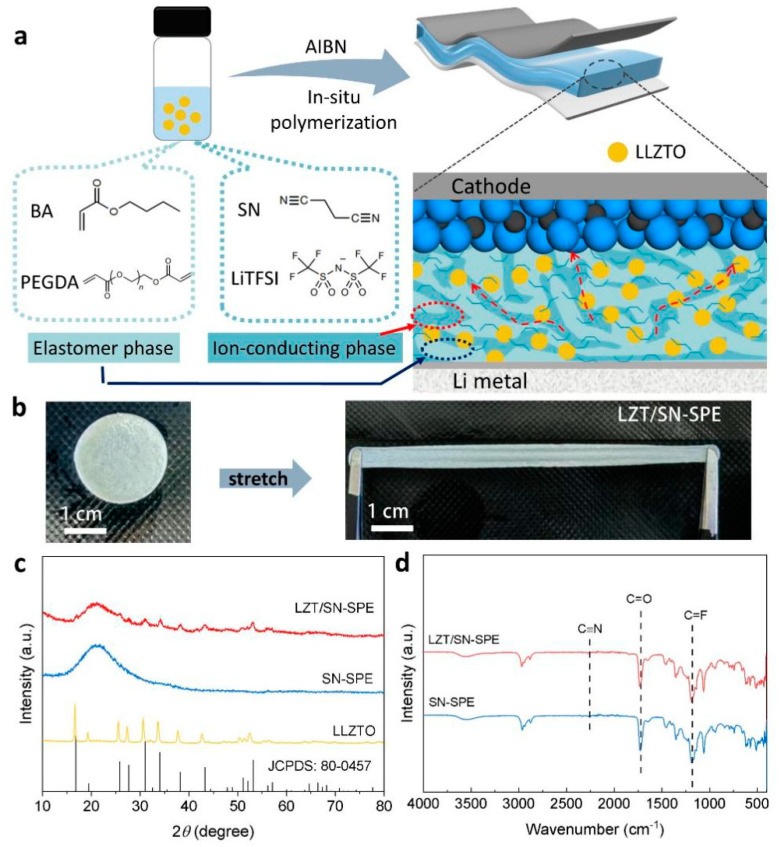
(**a**) Schematic illustration of the in situ preparation process for LZT/SN-SPE in solid-state LMBs. (**b**) Stretch digital picture of LZT/SN-SPE. (**c**) XRD patterns of LZT/SN-SPE, SN-SPE, and pure LLZTO. (**d**) FTIR spectra of LZT/SN-SPE and SN-SPE.

The LZT/SN-SPE is formed in the assembled LMBs using in situ polymerization as illustrated in Figure 1a. The precursor solution consists of BA monomers, SN plastic crystals, LLZTO ceramic, and LiTFSI. The high proportion of SN plastic crystals is selected as an ionic conductive material and an LLZTO ceramic fast Li^+^-ion conductor is selected as an inorganic filler. The weight ratio of the LLZTO ceramic particles in the LZT/SN-SPE is about 10%, as shown in Figure S1. AIBN and PEGDA are used as the thermal initiator and cross-linking agent, respectively. For polymerization, the precursor solution is dropped on the separator to assemble the LMBs and then heated at 70 °C for 2 h to realize in situ polymerization of the LZT/SN-SPE, which displays well-interfacial contact between the electrolyte and the electrodes. Owing to the existence of large butyl side groups of poly(butyl acrylate), the BA polymer is endowed with more free volume [44,45]. As a result, the LZT/SN-SPE shows ultra high elasticity and flexibility while keeping the functionality of the components (SN and LLZTO in our work), as shown in Figure 1b. 

The compatibility between LLZTO and SN-SPE is investigated by XRD and FTIR. Figure 1c shows that the XRD pattern diffraction peak positions of LZT/SN-SPE are well in accordance with LLZTO (JCPDS: 80-0457) and the BA/SN polymer matrix [46]. FTIR spectra (Figure 1d) illustrate the same characteristics of LZT/SN-SPE and SN-SPE. These results reveal that LLZTO has benign chemical compatibility with the BA/SN polymer matrix [43].

**Figure 2:**
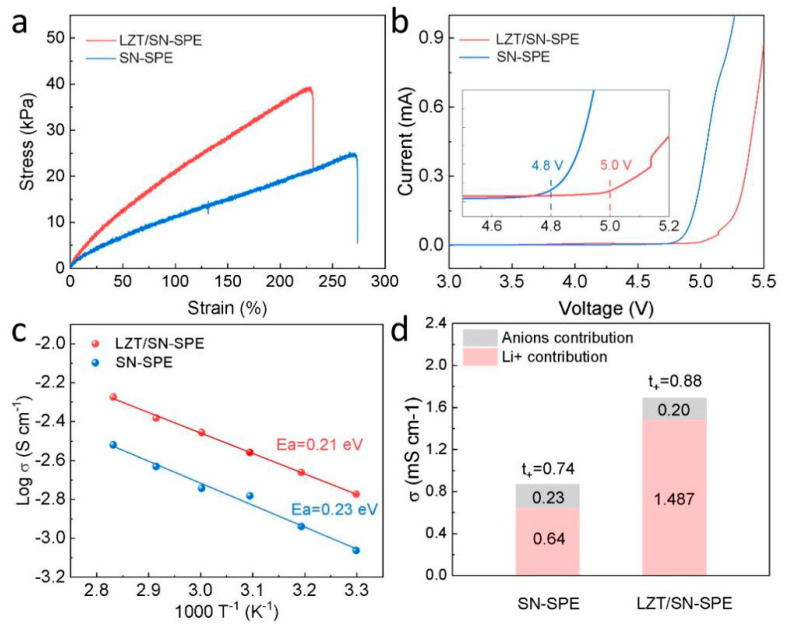
(**a**) Tensile stress-strain curves. (**b**) LSV curves with a scan rate of 1 mV·s^−1^ from 1.5 V to 6 V. Insets: enlarged profiles from 4.5 to 5.2 V. (**c**) Temperature-dependent ionic conductivity (30–80 °C) of LZT/SN-SPE and SN-SPE. (**d**) Ionic conductivity and Li^+^ transference number plot.

Good mechanical properties, such as tensile strength and elongation, are critical for solid electrolytes in LMBs. The typical stress–strain curve of the electrolytes in Figure 2a illustrates that the addition of the LLZTO ceramic filler strengthens the mechanical properties of the polymer matrix [16,47]. Both the SN-SPE and the LZT/SN-SPE exhibit outstanding elasticity with a strain of up to 273% and 232%, respectively. This indicates that the integration of the LLZTO ceramic filler did not compromise the mechanical robustness of the BA/SN polymer matrix. In addition, the oxidative stability is investigated by the LSV method at RT. The decomposition potential of SN-SPE is around 4.8 V, while that of LZT/SN-SPE is up to 5.0 V, as shown in Figure 2b. This indicates that LZT/SN-SPE exhibits a good electrochemical oxidation tolerance due to the high oxidation resistances of LLZTO, which can satisfy the requirement of high-voltage cathode materials [24]. The conductivity of electrolytes is further studied by temperature-dependent ionic conductivity measurements. The impedance under different temperatures is presented in Figure S2 and Figure 2a. The corresponding *E_a_* of LZT/SN-SPE and SN-SPE are 0.23 eV and 0.21 eV, respectively. The lower *E_a_* means a lower Li^+^ migration energy barrier, which is beneficial for the rapid Li^+^ migration in the electrolytes [48]. Moreover, Figure 2d, Figures S2 and S3 show the ionic conductivity and Li^+^ transference number (*t_+_*), which are important parameters for solid-state electrolytes. It can be seen that LZT/SN-SPE (1.69 mS·cm^−1^) has higher ionic conductivity than that of SN-SPE (0.87 mS·cm^−1^) at 30 °C (Figure 2d), indicating that the addition of LLZTO fillers can further increase the ionic conductivity of SN-SPE. The *t_+_* of LZT/SN-SPE is 0.88, which is higher than that of SN-SPE (0.74) and conventional PEO-based electrolytes (<0.5) [49]. The LLZTO particle fixe anion (TFSI^−^) and the polymer matrix can form a synergistic effect to restrict the movement of anions and lead to smooth Li^+^ migration, which is favorable to inhibit the growth of Li dendrites and boost the rate capacity [48,50,51]. 

**Figure 3:**
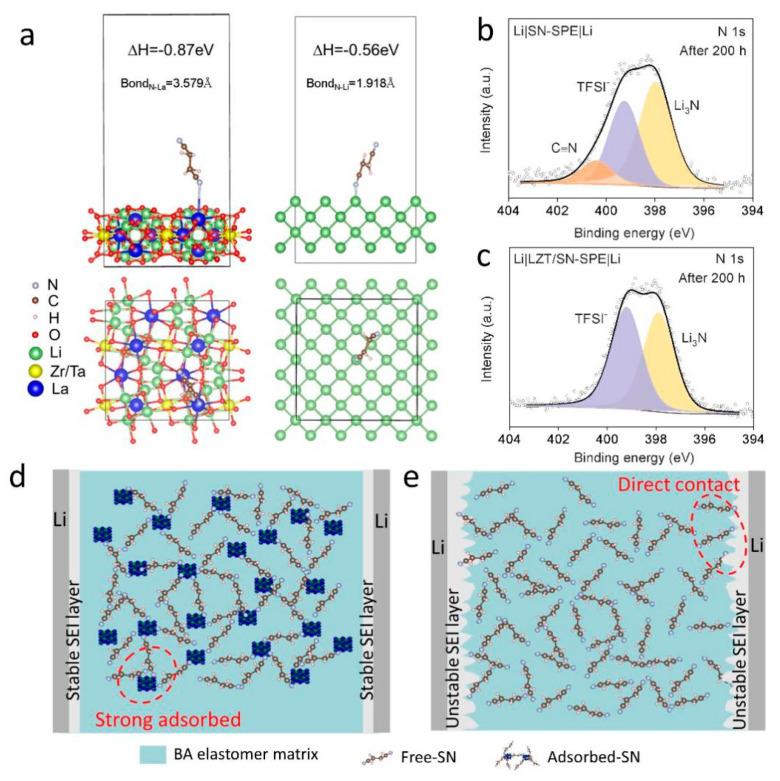
(**a**) The adsorption energy of SN molecule on LLZTO and lithium metal surfaces, respectively. XPS spectra of N 1s for the cycled lithium metal anode in (**b**) Li|SN-SPE|Li and (**c**) Li|LZT/SN-SPE|Li. (**d**,**e**) Schematics of Li|LZT/SN-SPE|Li and Li|SN-SPE|Li symmetrical cells during the Li plating and stripping.

A theoretical calculation is performed to reveal the role of the LLZTO filler in suppressing the side reactions between SN and the lithium metal anode. The interaction of SN on the surface of LLZTO and the lithium metal is calculated using first-principles calculations, as shown in Figure 3a. The adsorption energy of SN on the surface of LLZTO is −0.87 eV and is higher than that of SN and lithium metal (−0.56 eV). It illustrates that LLZTO has a stronger interaction with SN and plays a competitive role in the side reactions between SN and the lithium metal, which is in agreement with other research [14]. The –C≡N group prefers to bind with the La^3+^ cation in LLZTO than Li^+^ in the lithium metal. In addition, it was found using XPS analysis of the surface of lithium anode in cycled Li|SN-SPE|Li cells that mass C≡N groups can be observed on the lithium anode surface after 200 h cycles (Figure 3b), which root in SN. By contrast, the lithium anode surface of the Li|LZT/SN-SPE|Li cell is not found in the C≡N groups (Figure 3c), which further indicates that the addition of LLZTO could have “locked” free SN, inhibiting the side reaction with the lithium anode to a certain extent [14,43]. Therefore, with the assistance of LLZTO, free SN molecules inside the electrolyte can be “adsorbed and locked”, which is conducive to the suppression of the side reaction between SN and the lithium metal (Figure 3b). Without the assistance of LLZTO in the electrolyte, a direct interaction between the SN molecule and the lithium anode can cause serious side reactions and ultimately deteriorate the performance of the cells (Figure 3e). Hence, the integration of LLZTO ceramic particles can enhance the tensile strength and Li^+^ conductivity, improve Li^+^ transport kinetics of SN-SPE, and protect the lithium metal from SN attacks by “adsorbing and locking” free SN in SN-SPE. A stable and durable protective layer can be formed on the lithium anode, which is beneficial for the suppression of Li dendrites and for achieving excellent electrochemical performance.

In order to explore the stability between the lithium metal anode and the as-prepared LZT/SN-SPE, the Li plating/stripping behavior in the Li||Li symmetric cells is further evaluated. The Li|LZT/SN-SPE|Li symmetric cells show super long-term cycling stability over 1700 h at a current density of 1 mA cm^−2^ and a smooth overpotential at ≈±0.053 V, as shown in Figure 3a. As a comparison, the Li|SN-SPE|Li symmetric cells exhibit a relatively high overpotential of ≈±0.073 V and experienced a short circuit after 1550 h of cycling (Figure 3a). The lower overpotential and longer cycling stability of Li|LZT/SN-SPE|Li illustrates the superior interfacial compatibility between LZT/SN-SPE and the lithium anode and the uniform Li deposition behavior during the stripping–plating process. Figure S5 shows the evolution of EIS from 0 h to 60 h for cycling of the Li|LZT/SN-SPE|Li symmetric cell. The gradually stabilized charge-transfer resistance can also prove the formation of a stable and conductive SEI layer [28]. Moreover, even with the current density increasing to 5 mA cm^−2^, the Li|LZT/SN-SPE|Li symmetric cell can still be cycled stably for over 1100 h under a stable overpotential of 0.159 V. Hence, along with the integration of the LLZTO ceramic filler, the enhancement of the Li^+^ conductivity and transport kinetics of LZT/SN-SPE can realize excellent long term cycle stability, even at high current density. 

**Figure 4:**
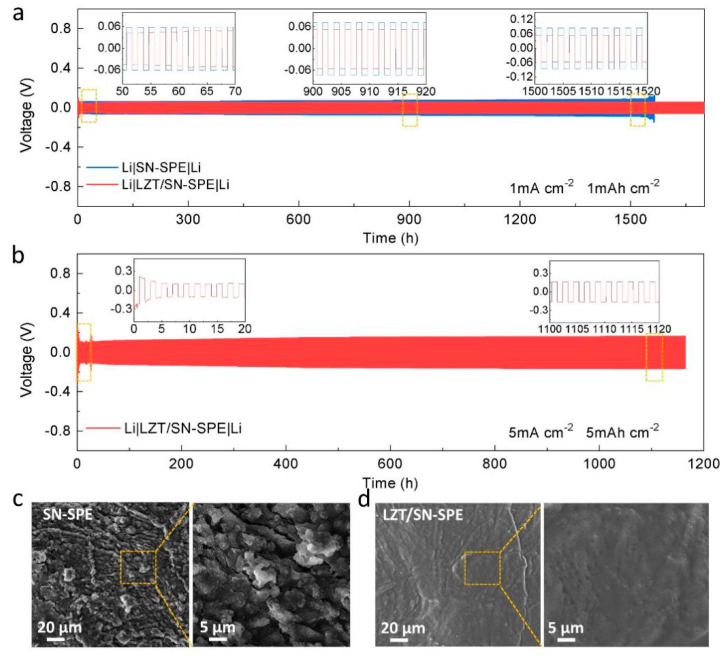
(**a**) Cycling performance of the symmetric Li cells with various electrolytes at 1 mA cm^−2^. (**b**) Cycling performance of the symmetric Li cells with LZT/SN-SPE at high current density. Insets: enlarged voltage profiles for different times. SEM images of the surface of lithium metal from the cycled (200 h) Li||Li symmetric cells with (**c**) LZT/SN-SPE and (**d**) SN-SPE.

In addition, post-mortem analysis is performed for the lithium metal of cycled Li||Li symmetric cells to better understand the excellent electrochemical performance of LZT/SN-SPE. After plating/stripping for 200 h of cycling, SEM images of the Li electrode surface corresponding to LZT/SN-SPE and SN-SPE display different morphologies, as shown in Figure 4c,d. The surface of the lithium metal for Li|SN-SPE|Li presents uneven protrusions and cracks. In contrast, the lithium metal surface for LZT/SN-SPE remains relatively smooth and flat without obvious dendrites or defects (Figure 4d), indicating that there is a uniform and durable SEI layer on the lithium anode surface. The main composition of the SEI layer for the cycled Li|LZT/SN-SPE|Li cells is further studied using XPS, as shown in Figure 3c and Figure S4. The results show that the main components of SEI are fast Li^+^ conductor Li_3_N and high mechanical strength LiF. These stable inorganic components contribute to the formation of a high durability and low resistance SEI layer, which inhibits the formation of Li dendrites, and achieves excellent electrochemical performance [43].

**Figure 5:**
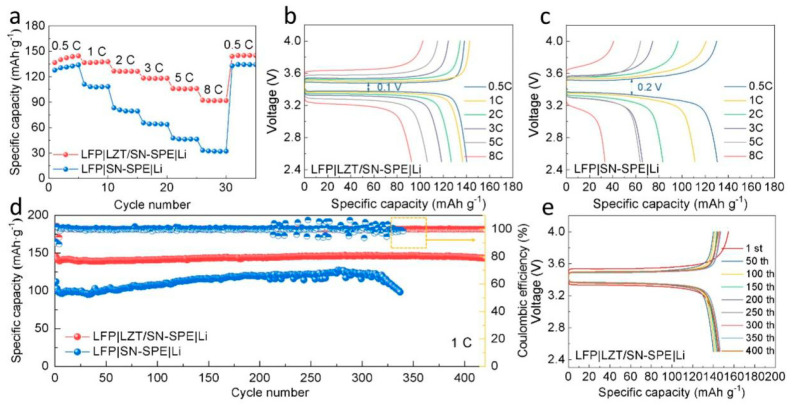
(**a**) Rate performance from 0.5 C to 8 C and (**b**,**c**) corresponding charge/discharge curves at different rates of LFP|LZT/SN-SPE|Li and LFP|SN-SPE|Li. (**d**) Long-term cycling stability of LFP|LZT/SN-SPE|Li and LFP|SN-SPE|Li at 1 C and (**e**) corresponding charge/discharge curves of LFP|LZT/SN-SPE|Li.

The electrochemical performance of the electrolytes in ASSLBs is also evaluated. The LFP||Li cells with LZT/SN-SPE and SN-SPE are assembled and tested at 30 °C. The rate performance of the two kinds of electrolytes is shown in Figure 5a. The LFP|LZT/SN-SPE|Li cell shows excellent rate capability with the specific capacities of 136.63 mAh g^−1^, 136.56 mAh g^−1^, 126.69 mAh g^−1^, 118.36 mAh g^−1^, 106.03 mAh g^−1^, and 92.40 mAh g^−1^ at the rates of 0.5 C, 1 C, 2 C, 3 C, 5 C, and 8 C (1 C = 170 mAh g^−1^), respectively. When the current density is returned to 0.5 C, the capacity can still reach 143.97 mAhg^−1^, indicating the excellent cyclic reversibility of LFP|LZT/SN-SPE|Li. By contrast, the LFP|SN-SPE|Li cell shows a poor rate performance, especially at high current density. The corresponding charge/discharge curves are shown in Figure 5b,c. The polarization voltages of LFP|LZT/SN-SPE|Li between charge and discharge are 0.1 V at 0.5 C, which is significantly lower than that of LFP|SN-SPE|Li (0.2 V at 0.5 C). The long and flat potential platform of LFP|LZT/SN-SPE|Li is still observed at 8 C, while the potential platform of LFP|SN-SPE|Li decays rapidly. The excellent rate performance and lower polarization voltage of LFP|LZT/SN-SPE|Li could be attributed to the increase in ionic conductivity and fast Li^+^ transport due to the addition of LLZTO ceramic particles [48]. Simultaneously, the long-term cycling stability of the electrolytes is evaluated at 1 C (Figure 5d) and the corresponding charge/discharge curves are shown in Figure 5e. The LFP|LZT/SN-SPE|Li delivers a higher initial discharge capacity of 145.31 mAh g^−1^ and shows outstanding cyclic stability over 420 cycles with a capacity retention ratio of 98.6% (capacity fade rate is 0.003% per cycle), which is advanced in the research of cells (Table S1) [7,14,16,23,24,41,48,52]. In contrast, the LFP|SN-SPE|Li observes a fluctuation after 200 cycles and has a rapid deterioration after 300 cycles. The advanced long-term cycling performance comes from the enhanced tensile strength mechanical properties of the LZT/SN-SPE and the suppression of the side reaction between SN and the lithium metal, which will form an intimate electrode–electrolyte contact and dense and stable SEI layer. 

## 4. Conclusions

In summary, a novel LZT/SN-SPE was prepared for the application of high-performance solid-state LMBs, which are based on an in situ-formed elastomer polymer matrix with ion-conductive plasticizer crystal SN and LLZTO nanoparticles embedded in it. The LZT/SN-SPE showed better performance in multiple aspects. Firstly, elastomers based on poly (butyl acrylate) have an excellent matrix that not only disperses functional components well but also maintains both mechanical elasticity and functionality. Secondly, the high proportion of SN plastic crystals in the elastic solid-state polymer electrolyte provided high ionic conductivity at room temperature. Thirdly, the incorporation of LLZTO increased the mechanical strength and electrochemical stabilization of the electrolytes, and then further improved the ionic conductivity and Li^+^ immigration. More importantly, benefiting from the strong coordination interaction between the N atom of SN and the La^3+^ cation in LLZTO, free SN molecules inside the electrolyte could be “adsorbed and locked”, which inhibited the side reaction between SN and the lithium metal to a certain extent. As a result, the LZT/SN-SPE obtained a high ionic conductivity of 1.69 mS cm^−1^ at 30 °C, a high Li-ion transference number (*t_+_*) of 0.88, and a favorable electrochemical window of about 5 V (vs. Li^+^/Li). Based on the LZT/SN-SPE, the Li||Li symmetric cells exhibited outstanding cycling stability of more than 1100 h at a high current density of 5 mA cm^−2^. Li||LFP cells achieved superior rate capability with a discharge capacity of 92.40 mAh g^−1^ at 5 C and long-term cycling with a 98.6% capacity retention after 420 cycles at 1 C.

## Data Availability

Data are contained within the article and Supplementary Materials.

## Supplementary Materials

The following supporting information can be downloaded at: https://www.mdpi.com/article/10.3390/nano14050433/s1, Figure S1: TG profiles of LZT/SN-SPE and SN-SPE; Figure S2: Electrochemical impedance curves of (a) LZT/SN-SPE and (b) SN-SPE at various temperature; Figure S3: Nyquist plots of the symmetric Li cells with (a) LZT/SN-SPE and (b) SN-SPE before and after polarization. Steady-state current measurement of the symmetric Li cells with (c) LZT/SN-SPE and (d) SN-SPE; Figure S4. XPS spectra of F 1s for the cycled lithium metal anode in Li|LZT/SN-SPE|Li; Figure S5. EIS evolution when cycling a Li|LZT/SN-SPE|Li cell at room temperature with a current density of 1 mA cm^−2^; Figure S6. Charge/discharge curves of LFP|LZT/SN-SPE|Li; Table S1 The electrochemical performance comparison of the LZT/SN-SPE with the other published works for ASSLBs.
