# Supplementary material for: In Situ Construction of Elastic Solid-State Polymer Electrolyte with Fast Ionic Transport for Dendrite-Free Solid-State Lithium Metal Batteries
Source: Nanomaterials (Basel). 2024 Feb 27;14(5):433. doi: 10.3390/nano14050433 (PMC10935166; doi:10.3390/nano14050433)
Supplement: Supplementary file 1 [file nanomaterials-14-00433-s001.zip › nanomaterials-2883389-supplementary.pdf]

Supplementary Materials:

Figure S1: TG profiles of LZT/SN-SPE and SN-SPE; Figure S2: Electrochemical impedance curves of (a) LZT/SN-SPE and (b) SN-SPE at various temperature; Figure S3: Nyquist plots of the symmetric Li cells with (a) LZT/SN-SPE and (b) SN-SPE before and after polarization. Steady-state current measurement of the symmetric Li cells with (c) LZT/SN-SPE and (d) SN-SPE; Figure S4. XPS spectra of F 1s for the cycled lithium metal anode in Li|LZT/SN-SPE|Li; Figure S5. EIS evolution when cycling a Li|LZT/SN-SPE|Li cell at room temperature with a current density of 1 mA cm^{-2}; Figure S6. Charge/discharge curves of LFP|LZT/SN-SPE|Li; Table S1 The electrochemical performance comparison of the LZT/SN-SPE with the other published works for ASSLBs.

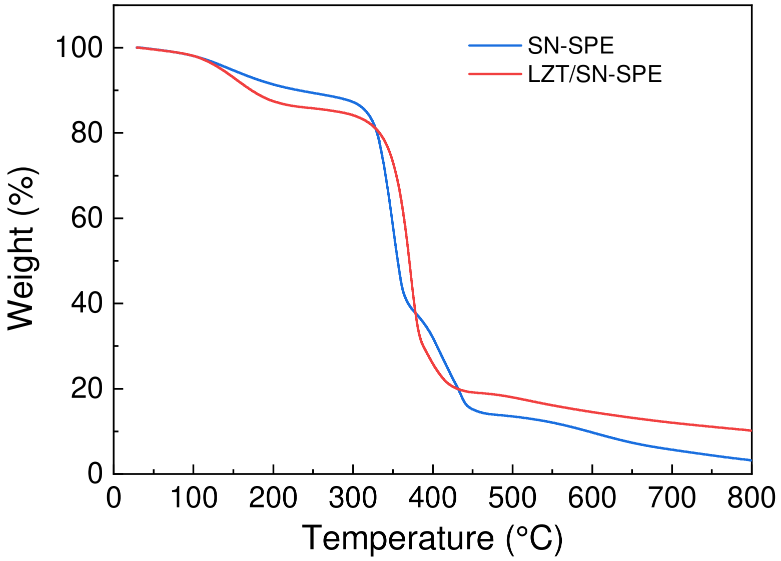

Figure S1. TG profiles of LZT/SN-SPE and SN-SPE

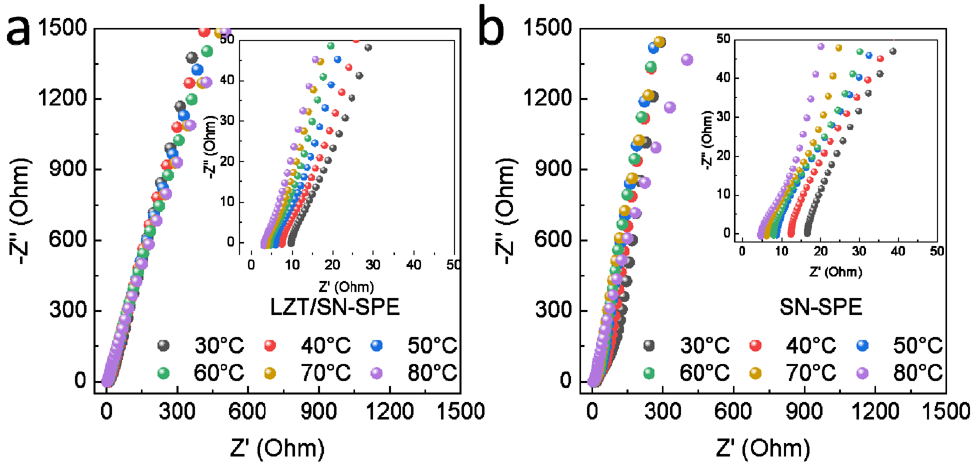

Figure S2. Electrochemical impedance curves of (a) LZT/SN-SPE and (b) SN-SPE at various temperature.

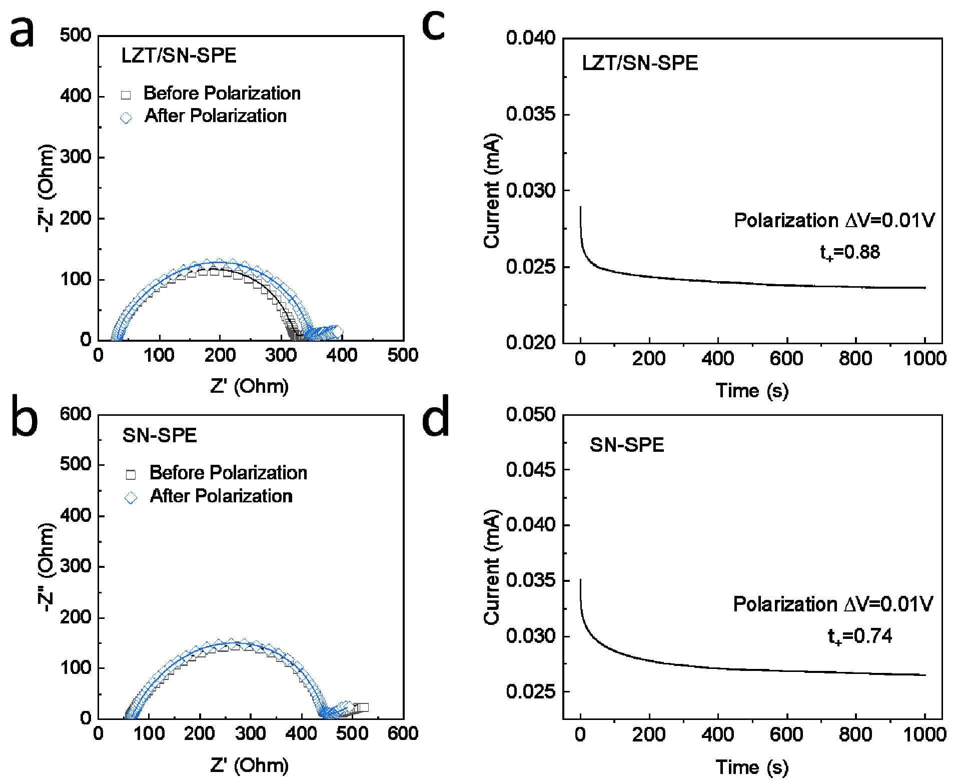

Figure S3. Nyquist plots of the symmetric Li cells with (a) LZT/SN-SPE and (b) SN-SPE before and after polarization. Steady-state current measurement of the symmetric Li cells with (c) LZT/SN-SPE and (d) SN-SPE.

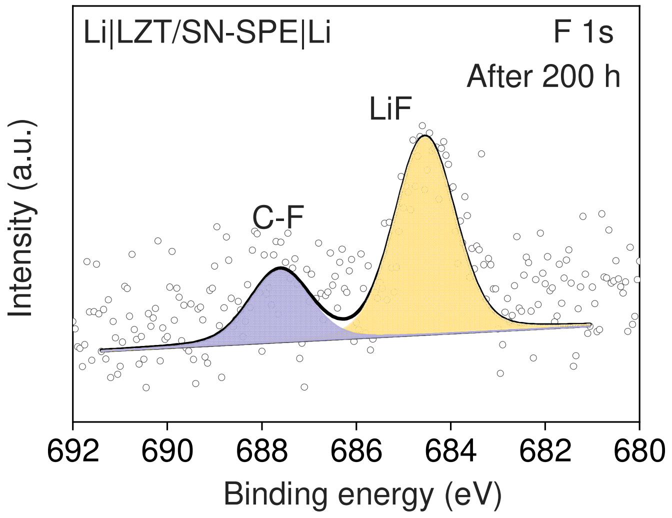

Figure S4. XPS spectra of F 1s for the cycled lithium metal anode in Li|LZT/SN-SPE|Li.

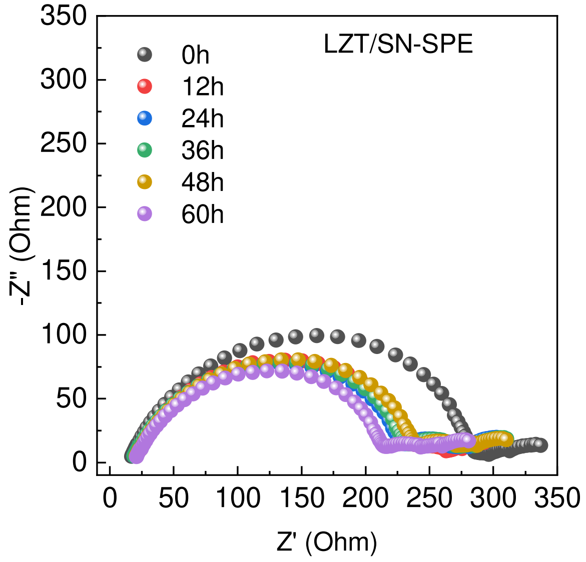

Figure S5. EIS evolution when cycling a Li|LZT/SN-SPE|Li cell at room temperature with a current density of 1 mA cm^{-2}.

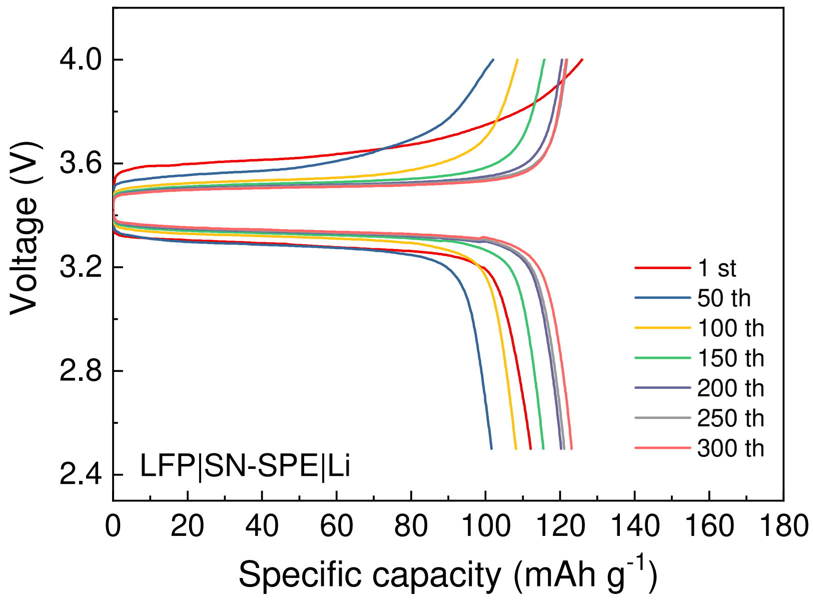

Figure S6. Charge/discharge curves of LFP|LZT/SN-SPE|Li.

Table S1 The electrochemical performance comparison of the LZT/SN-SPE with the other published works for ASSLBs.

**Ref.**	**Sample**	**Anode|Cathode**	**Cycle performance**	**capacity decay rate**	**Capacity (mAh g^{-1})**	**Rate(C)**
**Our work**	LZT/SN-SPE	Li|LFP	420 cycles, 98.6% at 1C	0.003%	136.63	0.5
					136.56	1
					126.69	2
					118.36	3
					106.03	5
					92.40	8
[7]	LiTFSI/SN/PTFE/LLZTO	Li|LFP	200 cycles, 90.3% at 0.2C	0.048%	157	0.1
					153	0.2
					142	0.3
					133	0.5
					115	1
[14]	LCPE-60	Li|LFP	240 cycles,	0.031%	157	0.1

			92.6% at 0.5C		153	0.2
					146	1
					140	2
					131	3
					86	5
[16]	IPLL-SSE	Li|LFP	200 cycles, 84% at 1C	0.08%	169.5	0.2
					160.5	0.3
					141.1	0.5
					125.8	1
					106.3	2
[23]	PVDF-HFP/SN/LiTFSI	Li|LFP	300 cycles, 98% at 0.5C	0.007%	162	0.1
					164	0.2
					160	0.5
					157	1
					148	2
[24]	LLZTO@SN/PAN	Li|LFP	500 cycles, 87% at 0.2C	0.026%	163	0.1
					160	0.2
					150	0.5
					126	1
					72	2
[41]	TPU/LLZTO/SN	Li|LFP	100 cycles, 88% at 0.5C	0.12%	168	0.1
					163	0.2
					150	0.5
					135	1
					121	2
					66	5
[48]	SN-CPE	Li|LFP	350 cycles, 88.3% at 1C	0.033%	160	0.1
					156	0.2
					155	0.5
					149	1
					133	2
[52]	PVDF–HFP-15%LLZO-LITFSI	Li|LFP	180 cycles, 92.5% at 0.5 C	0.042%	140	0.1
					124	0.2
					113	0.5
					100	1
					82	2
